# The FAK scaffold inhibitor C4 disrupts FAK-VEGFR-3 signaling and inhibits pancreatic cancer growth

**DOI:** 10.18632/oncotarget.1365

**Published:** 2013-09-30

**Authors:** Elena Kurenova, Jianqun Liao, Di-Hua He, Darrell Hunt, Michael Yemma, Wiam Bshara, Mukund Seshadri, William G. Cance

**Affiliations:** ^1^ Department of Surgical Oncology, Roswell Park Cancer Institute, Buffalo, NY; ^2^ Department of Pathology, Roswell Park Cancer Institute, Buffalo, NY; ^3^ Department of Pharmacology and Therapeutics, Roswell Park Cancer Institute, Buffalo, NY; ^4^ University of Florida, Gainesville, FL; ^5^ CureFAKtor Pharmaceuticals, Orchard Park, NY

**Keywords:** FAK Scaffold Inhibitor, pancreatic cancer, FAK-VEGFR-3 interaction, protein-protein interaction

## Abstract

Even with successful surgical resection and perioperative chemotherapy and radiation, pancreatic ductal adenocarcinoma (PDA) has a high incidence of recurrence. Tumor cell survival depends on activation of signaling pathways that suppress the apoptotic stimuli of invasion and metastasis. Focal adhesion kinase (FAK) is a critical signaling molecule that has been implicated in tumor cell survival, invasion and metastasis. We have previously shown that FAK and vascular endothelial growth factor receptor 3 (VEGFR-3) are overexpressed in cancer cells and physically interact to confer a significant survival advantage. We subsequently identified a novel small molecule inhibitor C4 that targeted the VEGFR-3-FAK site of interaction. In this study, we have shown that C4 disrupted the FAK-VEGFR-3 complexes in PDA cells. C4 treatment caused dose-dependent dephosphorylation and inactivation of the VEGFR-3 and FAK, reduction in cell viability and proliferation, cell cycle arrest and apoptosis in PDA cells. C4 increased the sensitivity of tumor cells to gemcitabine chemotherapy *in vitro* that lead to apoptosis at nanomolar concentrations of both drugs. C4 reduced tumor growth *in vivo*in subcutaneous and orthotopic murine models of PDA. The drug alone at low dose, decreased tumor growth; however, concomitant administration with low dose of gemcitabine had significant synergistic effect and led to 70% tumor reduction. Combination of C4 with gemcitabine had a prolonged cytostatic effect on tumor growth after treatment withdrawal. Finally, we report an anecdotal case of stage IV pancreatic cancer treated with gemcitabine in combination with C4 that showed a significant clinical response in primary tumor and complete clinical response in liver metastasis over an eight month period. Taken together, these results demonstrate that targeting the scaffolding function of FAK with a small-molecule FAK-VEGFR-3 inhibitor can be an effective therapeutic strategy against PDA.

## INTRODUCTION

Pancreatic ductal adenocarcinoma (PDA) is associated with a dismal prognosis with a 5 year survival of less than 5%. Chemotherapy and radiation treatment have had little impact on patient outcome, due to the presence of advanced disease at the time of diagnosis and high resistance to treatments, due to activation of redundant survival pathways in the tumor and stromal cells [1-3]. The therapeutic standard for metastatic disease has long been single-agent gemcitabine (GEM), which can improve quality of life in a subset of patients and moderately extend survival. Several studies investigating the role of conventional chemotherapy or targeted drugs in conjunction with GEM have been mostly unsuccessful [4]. The introduction of albumin-bound paclitaxel (nab-paclitaxel) affecting the desmoplastic stroma of PDA [3, 5] substantially improved median survival (6.8 vs. 12.2 months) [6]. Clearly, additional agents that address specific features of PDA are needed to improve outcomes in this aggressive disease. Many of the survival signals in PDA involve Focal Adhesion Kinase (FAK) [7, 8]. FAK, a non-receptor protein tyrosine kinase, localizes at focal adhesions and is a major regulator of the signals from ECM. It is one of the central molecules involved in regulation of cancer cell metastasis and survival and is associated with aggressive tumor behavior [9-11]. It was shown that in PDA there is a statistically significant correlation between FAK expression and tumor size, and FAK expression and tumor staging [12, 13]. Experimental data suggest that the aggressive capability of PDA is related to activation of FAK with subsequent activation of the Ras/Erk signaling pathway [7]. Indeed, FAK gene silencing suppressed anoikis resistance in PDA cells [14] and FAK siRNA potentiated gemcitabine-induced cytotoxicity *in vitro* and *in vivo* [15]. FAK has also been implicated in chemoresistance – FAK phosphorylation contributed to increased intrinsic chemoresistance to GEM in PDA cell lines [16]. These factors make it an important target in pancreatic cancer therapy. A few FAK kinase inhibitors were described [17] and it was shown that small molecule PF0562-271 reduced PDA tumor growth in orthotopic mouse model [18].

One of the most significant functions of FAK is its role as a scaffold for many growth-promoting proteins. FAK is involved in multiple protein-protein interactions and the scaffolding function of FAK plays a pivotal role in cancer cell signaling [19, 20]. Targeting cancer survival pathways with the drugs targeted to the scaffold is emerging as a promising novel approach [21]. Data on targeting specific protein-protein interactions of FAK demonstrate encouraging results in multiple cancer models, including PDA [22, 23]. One of the important components of the FAK scaffold is vascular endothelial growth factor receptor 3 (VEGFR-3 or Flt4). Previously, we have shown that FAK physically interacts with VEGFR-3 and provides important survival signals for breast cancer cells [24]. VEGFR-3 belongs to the VEGFR family of receptor tyrosine kinases and plays an important role in tumor vasculogenesis and angiogenesis [25-27]. Recent data demonstrate that lymphangiogenesis, facilitated by VEGFR-3 signaling, contributes to cancer dissemination [28, 29] and in PDA expression of VEGFR-3 ligands VEGF-C and VEGF-D has been shown to correlate with the rate of metastasis to lymph nodes [30, 31]. The VEGF-C, D/VEGFR-3 axis plays an important role in cancer cell proliferation, survival and resistance to chemotherapy [32-34]. We have shown that overexpression of VEGFR-3 increased aggressiveness of the cancer cells [35]. Increased VEGFR-3 level in pancreatic cancer tissues is related to marked expression in the cancer stroma and to moderate immunoreactivity in many cancer cells [30-32, 36]. Therefore VEGFR-3 upregulation on tumor blood vessels indicates a potential additional antiangiogenic effect for VEGFR-3 inhibitors [27, 36]. Indeed, inactivation of VEGFR-3 signaling by blocking antibodies, suppresses tumor growth by inhibiting tumor-induced neo-angiogenesis [25] and leads to both regression of the lymphatic network and to suppression of tumor lymph node metastasis [37, 38].

We have recently identified a novel molecular inhibitor C4 (chloropyramine hydrochloride), that targets the VEGFR-3-FAK site of interaction and disrupts the survival function of these two proteins [39]. C4 showed a marked reduction of breast tumor growth and was synergistic with doxorubicin chemotherapy in breast cancer xenograft models [39]. In this study we evaluated the effect of C4 on pancreatic cancer cells *in vitro* and pancreatic tumor growth *in vivo* in murine models of PDA and have shown its synergy with GEM in inhibition of pancreatic tumor growth. We report here an anecdotal case with stage IV pancreatic cancer treated with gemcitabine in combination with C4 that showed a significant clinical response in primary tumor and complete clinical response in liver metastasis over an eight month period.

## RESULTS

### C4 decreased the viability of pancreatic cancer cells, reduced phosphorylation of FAK and VEGFR-3 and decreased their complex formation

**Figure 1:**
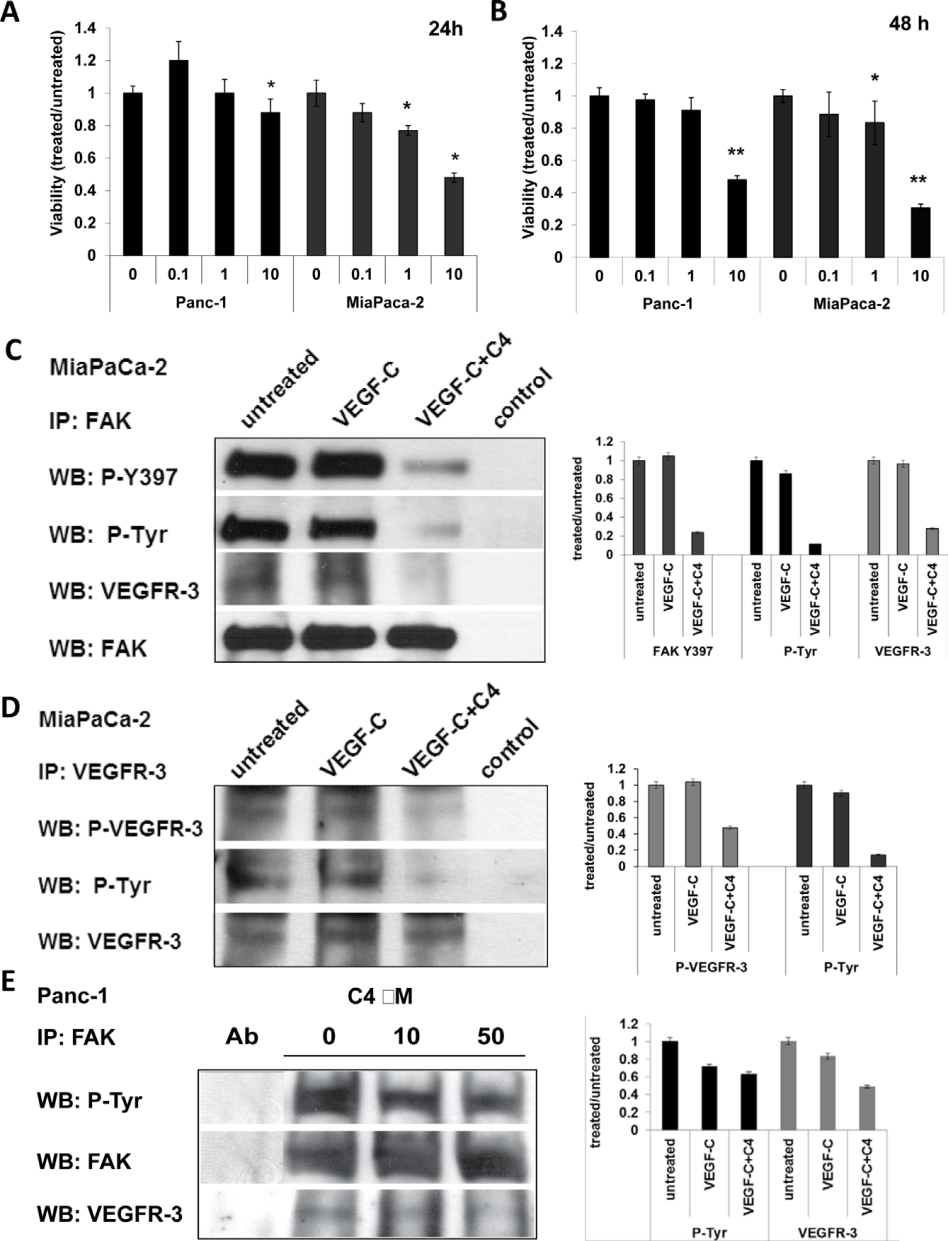
Compound C4 caused dose- and time-dependent decrease of viability of pancreatic cancer cells, dephosphorylation of FAK and VEGFR-3, and decrease of their complex formation

To determine the effects of C4 on pancreatic cancer cells, we first analyzed expression of FAK and VEGFR-3 in a panel of pancreatic cell lines and selected Panc-1 and MiaPaCa-2 for further analysis, based on the expression of both FAK and VEGFR-3 in these cells. Cells were treated with increasing concentrations of C4 and viability was measured after 24 and 48 h of treatment. Viability experiments showed that both cell lines were sensitive to C4 treatment and the effect was time- and dose-dependent (Figure 1A, B). MiaPaCa-2 cells were more sensitive to treatment with C4 than Panc-1 cells with up to 70% reduction of viability after 48 h of treatment (Figure 1A, B).

A, B. Compound C4 caused decrease of viability of pancreatic cancer cells (MTS assay). Cells plated on 96 well plates, grown 24 h and treated 24 h (A) and 48 h (B) with selected concentrations of small molecule C4. Data of MTS assay presented as ratio of OD treated to untreated cells, 1 corresponds to the 100% viability of the untreated cells. C. C4 dose-dependent dephosphorylation of FAK and VEGFR-3 is accompanied by decrease of FAK-VEGFR-3 association in PDA cells. Cells were treated with 10 μM (MiaPaCa-2) or 10 μM and 50 μM (Panc-1) of C4 for 24 h and phosphorylation of VEGFR-3 and FAK was analyzed by immunoprecipitation (IP) with consequent western blotting (WB) with pan-phospho-tyrosine antibody 4G10, P-VEGFR-3 (Tyr 1230/1231) specific antibody and antibody to major autophosphorylation site of FAK Y397. Control IP was done with isotype of the primary antibody. FAK precipitates were analyzed for the presence of VEGFR-3 protein with VEGFR-3 specific antibody. Densitometry was performed for each IP experiment and data are presented as graphs on the right panel of each figure.

Next we tested the phosphorylation status of FAK and VEGFR-3 after treatment with C4. Previously we have shown that C4 decreased both FAK and VEGFR-3 phosphorylation in breast cancer cells and inhibited complex formation. Therefore, the effects of C4 on phosphorylation were analyzed in FAK and VEGFR-3 immunoprecipitates of MiaPaCa-2 and Panc-1 cells after treatment for 24 h with increasing concentrations of C4. In MiaPaCa-2 cells, C4 treatment led to partial dephosphorylation of FAK on its major autophosphorylation site Y397, as well as additional FAK phosphorylation sites, that were revealed with anti-phospho-tyrosin antibody (Figure 1C). This decrease of the phosphorylated form of FAK occurred even in the presence of stimulating ligand VEGF-C. The VEGFR-3-FAK complex was significantly decreased in MiaPaca-2 cells after 24 h of treatment with 10 μM C4 (Figure 1C) suggesting that C4 disrupted the FAK-VEGFR-3 complex. Importantly, phosphorylation of VEGFR-3 in MiaPaCa-2 cells was also abolished even in the VEGF-C stimulated cells. This dephosphorylation affected the Tyr 1068 and Tyr1230/1231 sites in the VEGFR-3 kinase domain (Figure 1D). To assess changes in phosphorylation of FAK in Panc-1 cells FAK was immunoprecipitated from the lysates of cells treated with increasing concentration of compound C4. We analyzed precipitates with anti-phospho-tyrosine antibody, and found that 10 μM C4 treatment caused a decrease in total FAK phosphorylation, and 50 μM treatment considerably reduced the amount of active FAK (Figure 1E). We probed these precipitates for the presence of the VEGFR-3 protein and found that treatment with 10 μM C4 decreased the amount of VEGFR-3 co-precipitated with FAK and 50 μM C4 significantly abrogated complex formation. This finding correlated with the lower sensitivity of Panc-1 cells to C4 in the viability assay.

We have shown by kinase assays that dephosphorylation of FAK and VEGFR-3 after treatment with C4 was not related to C4 inhibition of their kinase activity or kinase activity of some other closely related kinases (Supplementary figure S1). Deactivation of FAK and VEGFR-3 should be associated with C4 inhibition of the FAK-VEGFR-3 binding site. We confirmed direct binding of small molecule C4 with the FAT domain of FAK in a direct binding assay *Bio-Layer Interferometry* (Supplementary figure S2). Thus compound C4 reduced the viability and specifically affected phosphorylation of FAK and VEGFR-3 and their complex formation in pancreatic cancer cells.

### C4 treatment caused G1 arrest, lead to apoptosis and affected Erk signaling pathway

**Figure 2:**
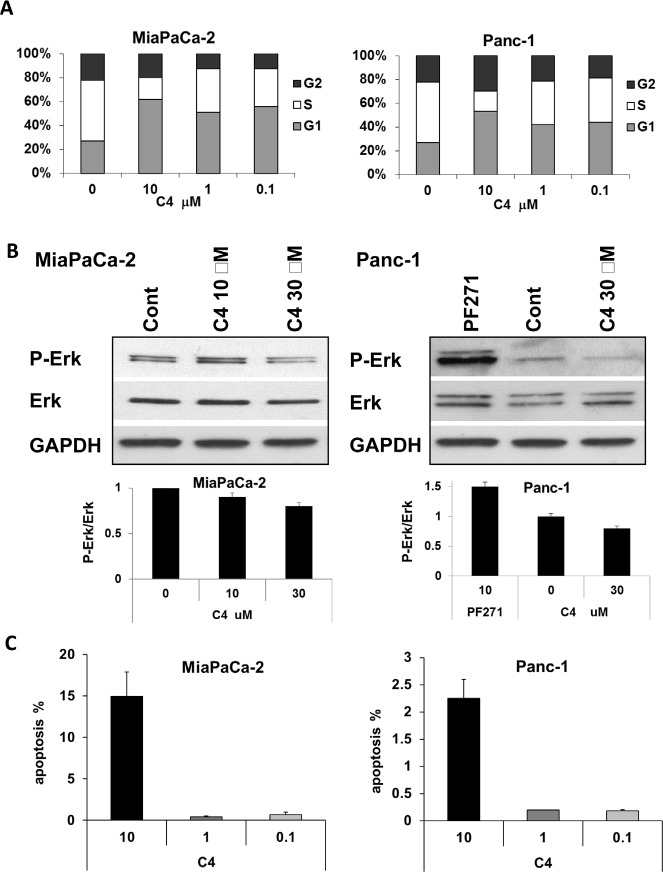
Treatment with C4 caused cell cycle arrest, dose-dependent apoptosis, and dephosphorylation of Erk1/2

Next we measured the *cell cycle* status of MiaPaCa-2 and Panc-1 cells after 24 h in the presence of increased concentrations of C4 (Figure 2A). Cell cycle distribution analysis showed that C4 delayed the cell cycle progression by arresting the cells in G_1_-phase in both cell lines. In MiaPaCa-2 cells, there was an increase in the percentage of cells in G_1_-phase from 27±1.1% (vehicle treated cells) to 61±2.1% (10 μM), with concomitant decrease in percentage of cells in S phase from vehicle treated 51±2.1% to 19±2.7% (10 μM) and a slight change in the percentage of cells in G_2_ phase. The Panc-1 cells started accumulating in G_1_-phase from 28±1% (vehicle treated) to 53±1.5% (10 μM), similar to MiaPaCa-2, with concomitant decrease in percentage of cells in S phase from vehicle treated 51±2.1% to 17±2.7% (10 μM) and a slight change in the percentage of cells in G_2_ phase. This accumulation of cells in G_1_ was seen even at a low C4 concentration of 100 nM in both cell lines.

A. Cell cycle analysis. Cells treated for 24 h with corresponding concentrations of C4 were analyzed with propidium iodide via flow cytometry. B. Effect of C4 treatment on activation of Erk1/2. Cells treated for 24 h with corresponding concentrations of C4 were analyzed with Erk1/2 and P-Erk1/2 (T202/Y204) antibody. PF271 is FAK kinase inhibitor PF-562271, used at 10 μM concentration. Densitometry was performed for each experiment and data are presented as graphs at the bottom of each figure. C. Detection of apoptosis. Cells treated for 24 h with corresponding concentrations of C4 were analyzed with Apo-Direct TUNEL Assay Kit via flow cytometry.

Two major pathways involved in the G_1_-to-S transition are the RAF-MEK-ERK and PI3-AKT pathways and it is known that FAK and VEGFR-3 are involved in the activation of these pathways. Hence, we determined the effect of C4 on Akt and Erk activation. We found that C4 did not affect Akt phosphorylation at a 24 h time point, with selected concentrations (data not shown). At the same time C4 had a dose-dependent effect on Erk dephosphorylation in MiaPaCa-2 cells (Figure 2B, left panels) with significant inhibition of Erk1/2 at higher concentration. In drug resistant Panc-1 cells we compared the effect of C4 with FAK kinase inhibitor PF-562271 (PF271) and found that 24 h 10 μM treatment with C4 also led to dephosphorylation/deactivation of Erk1/2. At the same time 10 μM PF271 caused activation of Erk1/2 (Figure 2B, right panels) with Erk phosphorylation increased 1.5 times.

We next investigated whether the C4-mediated decrease in viability of MiaPaCa-2 and Panc-1 cells was due to apoptosis, because decrease in FAK phosphorylation in cancer cells usually coincides with cell death [40, 41]. We found that exposure for 24 h to 10 μM C4 induced apoptosis in MiaPaCa-2 to higher extend than in Panc-1 cells (Figure 2D) and that corresponds to higher C4 resistance of Panc-1 found in viability test. We concluded that C4 caused PDA cell death through G_1_ cycle arrest and through decrease the survival ability of the cells by dephosphorylation/deactivation of Erk1/2.

### C4 sensitized pancreatic cancer cells to cytotoxic therapy *in vitro* at nanomolar concentrations

**Figure 3:**
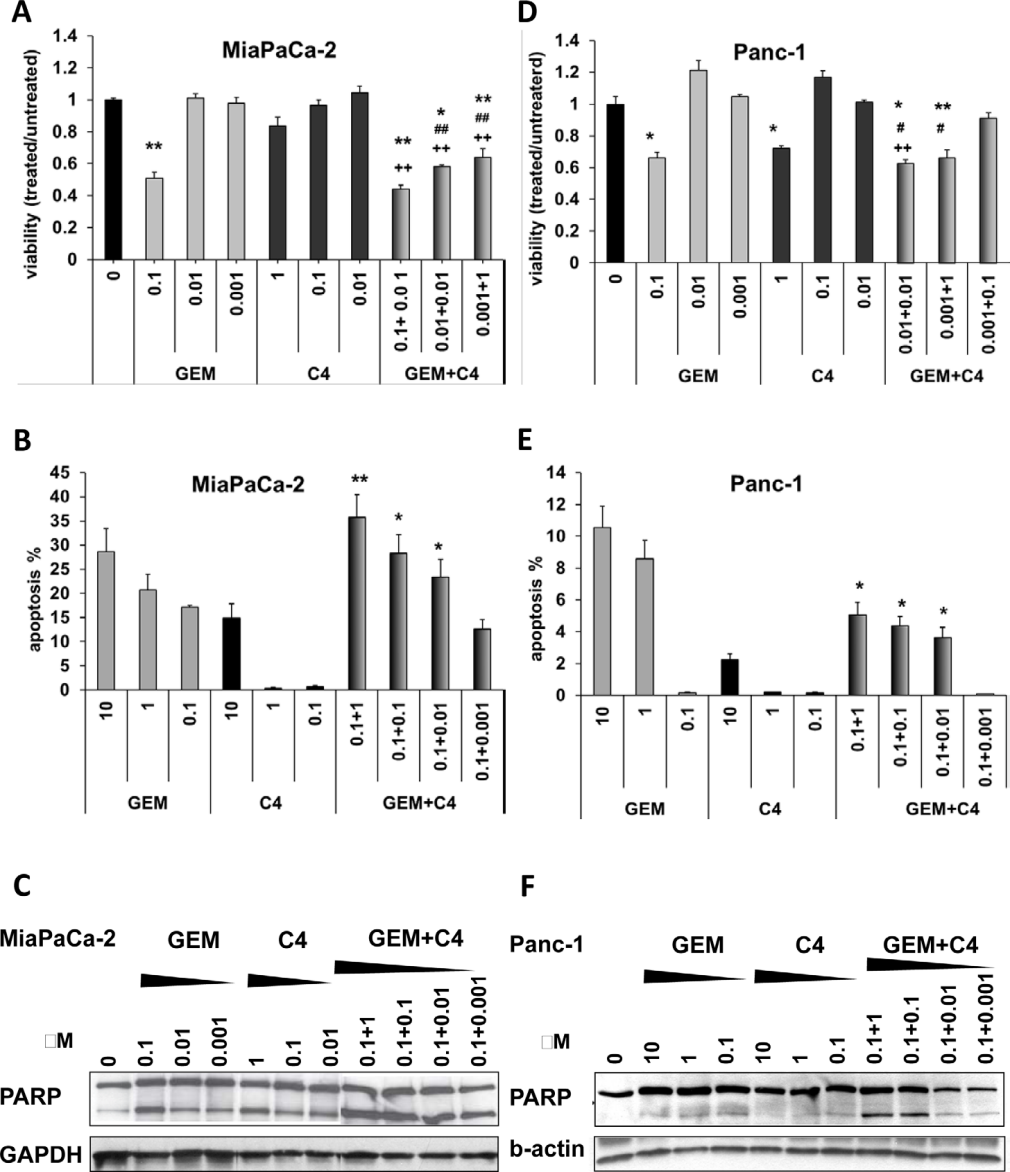
C4 sensitized pancreatic cancer cells to chemotherapy treatment *in vitro* and caused synergistic effect leading to apoptosis at nanomolar concentrations

Our fundamental hypothesis is that disrupting of the FAK scaffold-dependent survival pathways will augment tumor sensitivity to cytotoxic therapy. We tested this hypothesis by combining C4 with standard gemcitabine (GEM) treatment in PDA cells and analyzed the effect at reduced doses of both drugs. In MiaPaCa-2 cells, viability was not affected by C4 or GEM at low doses of 10 nM or 1 nM (Figure 3A). However, when 10 nM doses of both C4 and GEM were combined there was a decrease in the cell viability by 40%. Furthermore, combination of C4 with GEM caused dose-dependent apoptosis and at nanomolar doses of both drugs this effect was synergistic (Figure 3B). This finding was confirmed biochemically with increasing PARP cleavage in case of dual treatment (Figure 3C). Although the Panc-1 cells were more resistant to GEM treatment, there was a similar decrease in viability of approximately 40% with a combination of 10 nM of each drug (Figure 3D). TUNEL analysis of Panc-1 cells confirmed that they were more resistant to apoptosis caused by GEM and C4 and only a small portion of the cells underwent apoptotic cell death even at 10 μM of each drug. However, we also saw an increase in the number of apoptotic cells after combined treatment and confirmed TUNEL data biochemically by demonstration of PARP cleavage in Panc-1 cells (Figure 3F). Therefore, a statistically significant decrease in cell viability in comparison with control and each drug alone was found at doses of 10 nM for both C4 and GEM. These results demonstrated that disruption of FAK-VEGFR-3 protein-protein interaction with small molecule C4 caused apoptosis in pancreatic cancer cells and synergized with cytotoxic treatment, leading to increased apoptosis at nanomolar concentrations of both drugs.

Decreased viability and proliferation and increased apoptosis (TUNEL assay, flow cytometry) in pancreatic cancer cells after dual treatment C4 plus gemcitabine (GEM) for 24-72 h at low nanomolar doses. A. MTS assay, B. TUNEL flow cytometry and C. Western blot analysis in MiaPaCa-2 cells. D. MTS assay, E. TUNEL flow cytometry and F. Western blot analysis of Panc-1 cells. Degradation of PARP was detected. All concentrations - μM. * P<0.05 and ** P<0.01 relative to control, + relative to C4 alone, # relative to chemotherapy alone.

### C4 decreased tumor growth *in vivo* and sensitized to conventional chemotherapy

**Figure 4:**
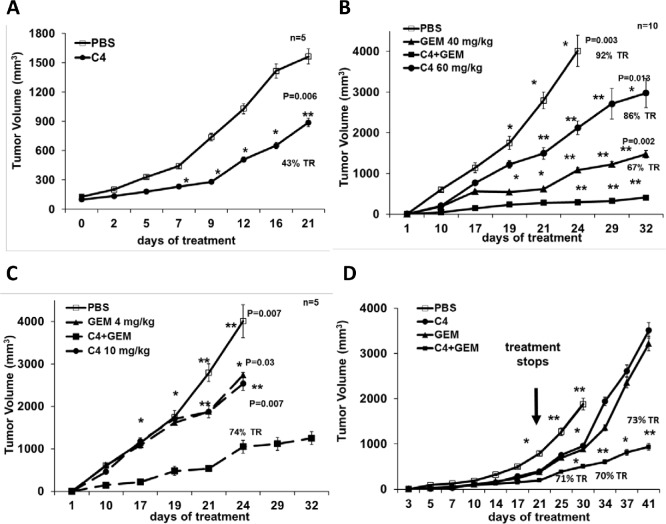
C4 reduced tumor growth *in vivo* in mouse model of pancreatic cancer as single agent and in combination with gemcitabine (GEM)

Next we examined the activity of C4 against PDA using the MiaPaCa-2 xenograft model. Intraperitoneal injections of C4 (50 mg/kg) were started either the next day after the cells were injected (prevention model) or when the tumors reached on average a size of 100 mm^3^(intervention model). After 21 days of C4 treatment, the tumor volume of established tumors was significantly smaller than the tumor size in control group (Figure 4A), with approximate tumor growth reduction of 43% (P=0.006) (Figure 4A). These data have shown that small molecule C4 was able to reduce tumor growth *in vivo*, as a single agent. Nonetheless, we recognized that effects of C4 on tumor growth were moderate and we anticipated use of this inhibitor in combination with cytotoxic agents. Indeed, concomitant administration of C4 (50 mg/kg 1xq) with GEM (40 mg/kg 1x4d) reduced tumor growth more than 90 % (P=0.003) in a MiaPaCa-2 prevention model (Figure 4B). This result prompted us to confirm the chemotherapy sensitizing effect of C4 using low concentrations of GEM and C4 in our next experiment. When GEM dose was reduced 10 fold (4 mg/kg) its effect on tumor growth as a single agent was decreased from 73% to 32% of tumor growth reduction (Figure 4C), and reduced concentration of C4 (10 mg/kg), led to 30% of tumor growth reduction. However, combination of C4 and GEM at these concentrations caused more than 70% tumor growth reduction (P=0.007). We concluded that C4 increases the sensitivity of PDA cells to chemotherapy and the therapeutic dose of the drug can be reduced.

Female nude mice were subcutaneously inoculated with MiaPaCa-2 cells. A. C4 treatment of established tumors. Intraperitoneal injections (IP) with compound C4 (60mg/kg, daily). TR – tumor growth reduction relative to control. B. Treatment with high concentrations of C4 and GEM, IP with compound C4 (60mg/kg, daily), GEM (40 mg/kg, Q4dx6) and combination were started next day after cell injection. TR – tumor growth reduction in “dual treatment” group relative to control at day 24 and to C4 treatment group at day 32. C. Combination of C4 with GEM at low concentration had synergistic effect on tumor grows. Treatment with low 10 mg/kg dose of C4 daily alone or in combination with GEM low 4 mg/kg once every 4 days (IP) was performed for 24 days. TR – tumor growth reduction in “dual treatment” group relative to control at day 24. D. Combination of C4 with GEM has prolonged cytostatic effect on tumor growth after treatment withdrawal at day 21. TR – tumor growth reduction in “dual treatment” group relative to control at day 30 and to C4 treatment group at day 41.

### Combination of C4 with Gemcitabine had prolonged cytostatic effect on tumor growth after treatment withdrawal

Next we compared tumor re-growth after treatment withdrawal. Mice were injected with C4 for 21 days and then treatment stopped, but we continued to measure the size of the tumors for the next 21 days. We found that tumors treated with a single drug re-grew much faster than tumors treated with combination of the drugs (Figure 4D). The size of the tumors in the group with combination of C4 and GEM was approximately 30% of the size of the tumors from groups with C4 and GEM alone and this difference was statistically significant.

**Figure 5:**
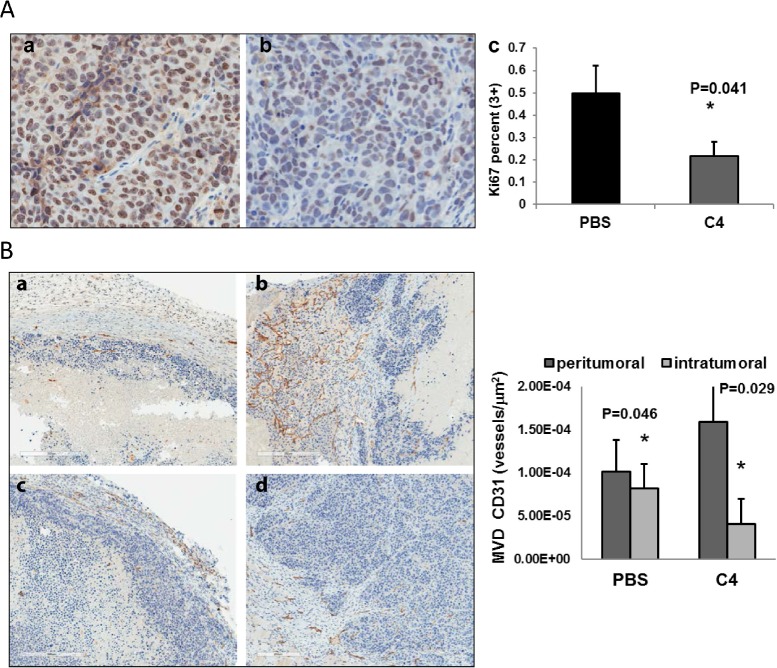
A

All tumors were analyzed by IHC with Ki67 antibody and staining confirmed decrease in proliferation of tumor cells (Figure 5A). We also analyzed the tumors for the expression of vessel marker CD31 and measured vessel density, because both tyrosine kinases FAK and VEGFR-3 are important for vasculogenesis and are expressed on endothelial cells. We found that vessel density was changed differently in tumor periphery and inside the tumors. The peritumoral vessel density was significantly increased (Figure 5B, compare a and c). At the same time we saw a decrease in the intratumoral vessel density after four weeks of treatment with C4 (Figure 5B, compare b and d). And we found that this vessel density reduction was mostly related to vessel size reduction and normalization of vessel network.

Proliferation of tumor cells was reduced in MiaPaCa-2 xenograft tumors after four weeks of treatment with C4. Staining with Ki67 antibody. Panel a – representative tumor from control PBS treated group, panel b – tumor from C4 treated group. Panel c – percentage of Ki67 highly (3+) stained nuclei in PBS and C4 treated tumors. B, C. C4 affects vessel formation in xenograft tumors. B. Tumors immunohistochemistry with mCD31 antibody. Panels a and b - representative tumors from control PBS treated group, panels c and d – tumors from C4 treated group. Vessel density analysis: peritumoral vessel density was significantly increased (dark grey bars on the graph) and intratumoral vessel density was significantly reduced after four weeks of treatment with C4 (light grey bars). (MVD - mean vessel density).

### C4 reduced tumor growth *in vivo* in mouse orthotopic model of pancreatic cancer

**Figure 6:**
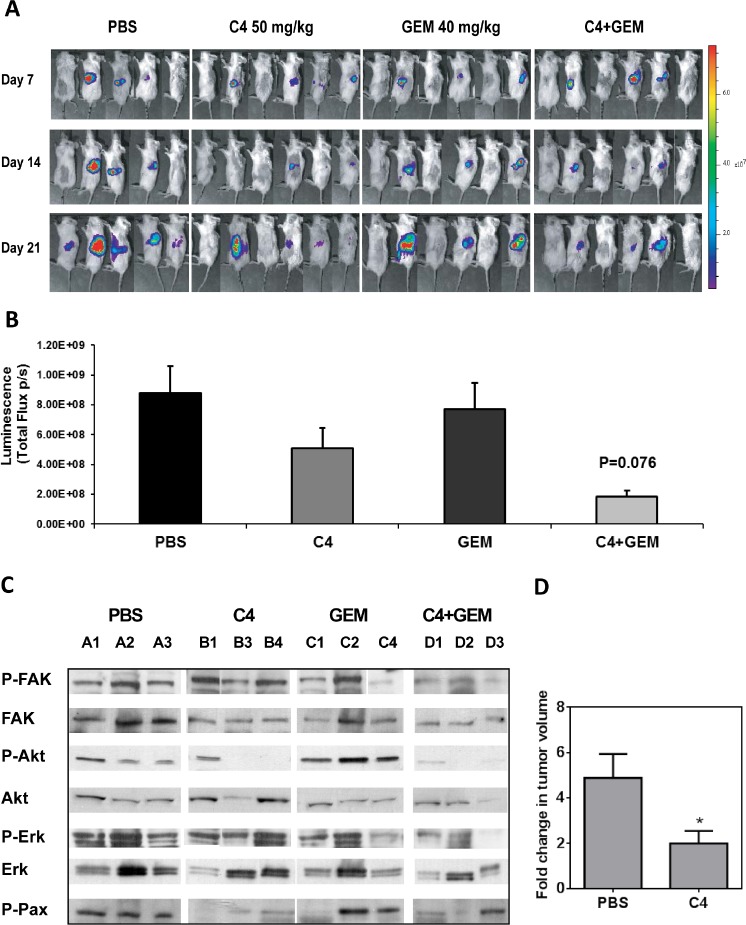
C4 reduced tumor growth *in vivo* in mouse orthotopic model of pancreatic cancer

An orthotopic model of pancreatic cancer was used to assess the effect of C4 on tumor growth at orthotopic location and spread of metastases. MiaPaCa-2 cells, transfected with luciferase expressing plasmid, were directly injected into the pancreas (6 mice per group). Treatment with combination of C4 and GEM was initiated one week after tumor cell inoculation and continued for 21 days; while tumor growth was monitored by bioluminescence imaging (Figure 6A). Maximal reduction in tumor burden was observed in the dual treatment group. These tumors were smaller than tumors in control group, though the difference did not reach statistical significance (P=0.076) (Figure 6B). Nevertheless, phosphorylation of FAK, Akt, Erk and FAK substrate Paxillin was reduced not only in combination C4 plus GEM treatment group but in C4 group too (Figure 6C).

A. Orthotopic MiaPaCa-2 xenografts established in SCID mice. 1x10^6^ MiaPaCa-2 cells, stably transfected with Luciferase expressing plasmid, were injected into the pancreas of SCID mice (n=6). Bioluminescence (BL) imaging was performed at days 7, 14 and 21 after tumor cell injection. B. Tumor burden (measured by BLI; photon flux) at the end of the experiment. C. Western blot of tumor lysates probed with P-FAK (Y397), P-Akt (S473), P-Erk1/2 (T202/Y204) and P-Paxillin (Y118) antibody. D. Orthotopic model with Human Pancreatic Tumor 11424. Mice treatment with C4 started on day 42 post implantation and followed for 21 days. MRI was performed once a week after day 20 post implantation. Bar graph shows fold change in tumor volume (measured by MRI) in PBS and C4-treated animals at 3 weeks post treatment compared to baseline pretreatment estimates.

Finally we examined the activity of C4 against patient tumor-derived pancreatic xenografts. For this study, after 42 days of growth, tumor bearing mice (n=8 per group) were treated with either PBS or C4 (50 mg/kg, IP) for 21 days. Non-invasive magnetic resonance imaging was performed before and after treatment to monitor primary tumor growth and presence of metastases. T2-weighted MRI was performed to assess tumor volume in animals at baseline and following three weeks of therapy (Figure 6D). Primary tumor volumes of animals in both groups were comparable at the time treatment was initiated (PBS = 527 ± 129 mm^3^, C4 = 724 ± 148 mm^3^; p>0.05). A significant increase (p = 0.004) in primary tumor volume was observed in PBS-treated controls over the three week period (2104 ± 378 mm^3^). In contrast, C4-treated animals did not show any significant increase in tumor volume on day 21, compared to baseline. Compared to >4-fold increase in tumor growth seen in controls, C4-treated animals showed a two-fold increase in tumor growth (p = 0.03). At the end of the experiment, MRI revealed the presence of liver and lung metastases in 2/8 mice (25%) in the PBS treated group. No evidence of metastasis was seen in C4 treated mice.

### Potential for clinical application

**Figure 7:**
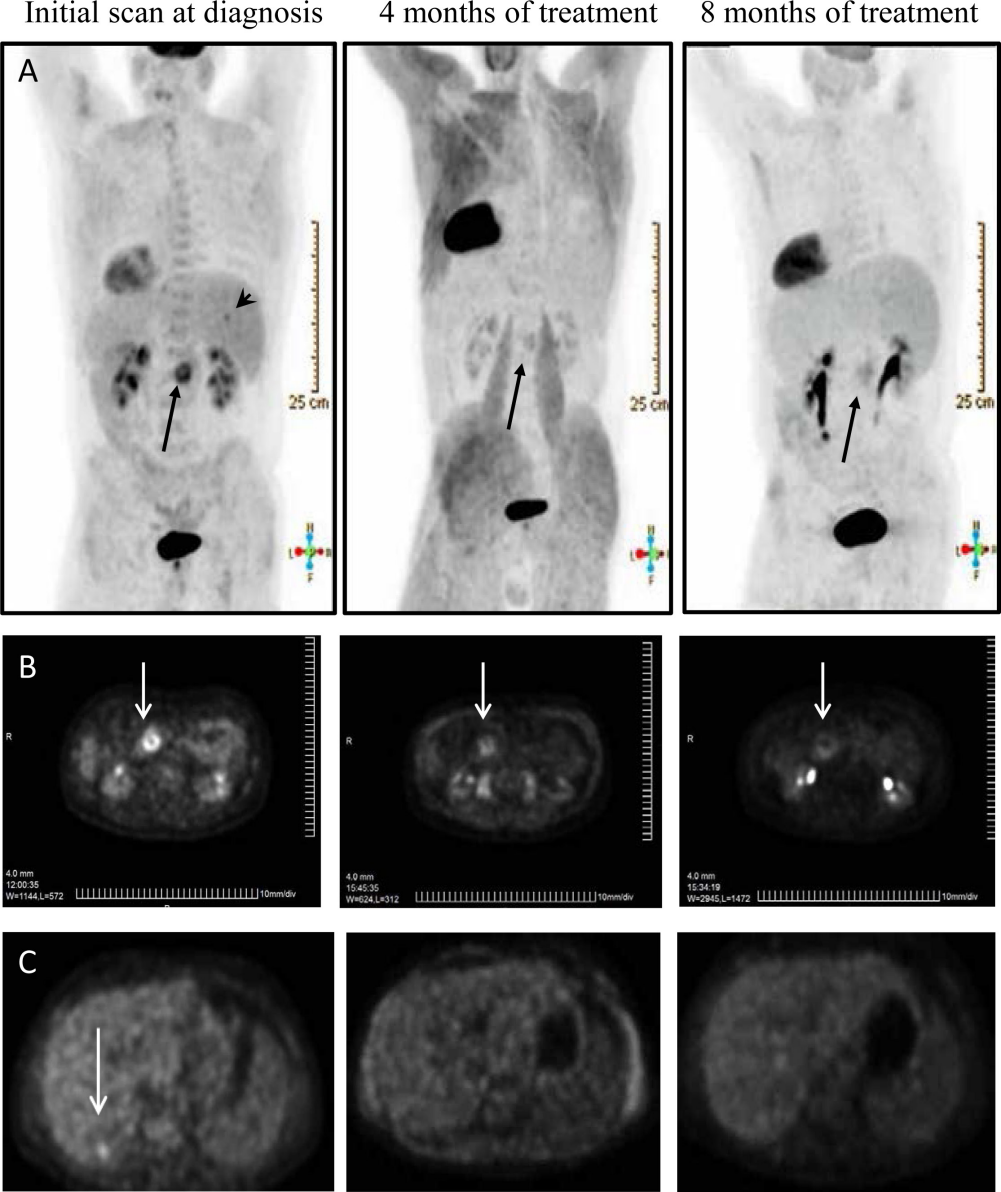
Prolonged regression of stage IV pancreatic cancer (primary tumor and liver metastasis) treated with C4 in combination with gemcitabine

Compound C4 is a small molecule chloropyramine hydrochloride known as a competitive reversible H1-receptor antagonist, widely used in Eastern Europe and available over the counter. Figure 7A CT scan image of a patient with stage IV pancreatic cancer who personally elected to take daily oral chloropyramine hydrochloride, in parallel with the standard course of gemcitabine chemotherapy. Figure 7B shows significant clinical response of primary tumor over an eight month period of dual treatment. Importantly, liver metastasis seen on initial CT and PET scan, has shown a complete clinical response over an eight month period (Figure 7C).

A. Axial PET scans show liver metastasis (arrowhead) on initial scan, undetectable 4 and 8 months after treatment with C4 and chemotherapy. Primary tumor (arrow) shows marked response to treatment with minimal PET activity. Scans oriented to view patient from behind (liver on right side). B. PET scans show sequential response of advanced pancreatic cancer after treatment with C4 and chemotherapy. The primary tumor in the pancreas (arrow) shows sustained regression over 8 months of treatment. C. PET scan at diagnosis of Stage IV pancreatic adenocarcinoma demonstrates liver metastasis (arrow). Scans demonstrated a complete clinical response after 4 and 8 months of treatment with C4 and chemotherapy.

## DISCUSSION

Pancreatic ductal adenocarcinoma is known as aggressive disease [42] with poor prognosis. Clinical development of targeted agents directed against the well-defined EGF/EGF receptor axis, the mutant KRAS protein, MMPs, and VEGF-mediated angiogenesis, alone or in combination with gemcitabine-based standard chemotherapy, are not very efficient. Therefore, targeting relatively unexplored signaling pathways can increase our understanding of the complex biology of pancreatic cancer and provide new therapy. In this study we explored the simultaneous targeting of pathways related to the FAK and VEGFR-3 signaling in PDA. Using the FAK inhibitor C4 targeted to VEGFR-3 protein-protein binding site on the FAK FAT domain, we examined the effect of C4 on PDA cell biology *in vitro* and therapeutic efficacy *in vivo*.

FAK plays a significant role in the regulation of adhesion turnover and migration and is critical for the survival and growth of the cancer cell [43, 44]. Consistent with this, we have shown that the treatment with C4 of MiaPaCa-2 and Panc-1cells inhibited PDA cell viability, proliferation and migration. Importantly, we confirmed direct binding of C4 (chloropyramine) to the FAT domain of FAK with Octet biolayer interferometry and confirmed high specificity of C4 actions. C4 affects FAK-VEGFR-3 complex formation and phosphorylation of these proteins but does not affect phosphorylation or kinase activity of closely related proteins. Analysis of phosphorylation status of FAK and VEGFR-3 revealed a decrease in total phosphorylation and in case of VEGFR-3 - dephosphorylation on Tyr-1230 and Tyr-1231. Phosphorylation of these sites is important for interaction with Grb2 and subsequent activation of the Akt1 and Erk1/2 signaling. Tyr-1230/1231 contributes, together with Tyr1337, to proliferation, migration, and survival of endothelial cells [45]. We found that phosphorylation of Grb2 dramatically reduced in PDA cells treated with C4 (not shown) and activation of Akt and Erk is inhibited.

We have shown that C4 treatment caused apoptosis of PDA cells through G_1_ cycle arrest and through the decrease the survival ability of the cells by dephosphorylation/deactivation of Erk. This finding correlates well with published literature on the involvement of FAK in cell cycle regulation via activation of protein kinase C isoforms and cyclins [46].

Our *in vitro* analysis demonstrated significant effect of C4 on proliferation and death of PDA cells as a single agent. Furthermore, its combination with GEM demonstrated synergistic effect on cell viability and induction of apoptosis even at low nanomolar concentrations of both drugs, ineffective alone. This effect was confirmed in our mouse model, where C4 or GEM alone reduced tumor growth to a significantly low extent than a combination of these two drugs. In accordance with *in vitro* data, ten times lower concentration of gemcitabine (4 mg/kg) in combination with six times lower concentration of C4 (10 mg/kg) reduced tumor growth almost as efficiently as combination of high concentrations (TR=74% vs. TR=92%). Surprisingly, our results differ from the preclinical study of FAK kinase inhibitor PF-562,271, where combination with GEM did not increase tumor growth inhibition [18]. Our comparison of schedule and doses revealed very similar treatment approach (60 mg/kg IP once a day for C4 vs. 33 mg/kg twice a day for Pfizer inhibitor PF-562271). We hypothesized that FAK inhibitors decrease pro-survival function of FAK and make cancer cells more vulnerable to additional stress, but this discrepancy in data suggests that inhibition of kinase function of FAK with kinase inhibitor affects different mechanisms/pathways in cancer cell than PPI inhibitor. In addition, we have shown an anecdotal report of a patient with stage IV pancreatic adenocarcinoma who had a clinical complete response to C4/gemcitabine based therapy.

Targeting the site of protein-protein interaction represents a novel approach to FAK inhibition with direct disruption of downstream signaling. Protein-protein interactions are now becoming increasingly attractive targets for cancer therapeutics [47, 48]. Within the last few years, sufficiently effective small-molecule inhibitors have been identified for a few important PPIs [21]. In addition to well-known nutlins, new inhibitors are entering clinical studies and more are at preclinical stage. Thus, allosteric FAK inhibitor Y15, targeting major autophosphorylation site of FAK Y397, which affects not only FAK kinase activity but, more importantly, FAK interactions with Src and PI3K, showed promising results in treatment of breast, pancreatic, colon and glioblastoma cancers [49]. PPI inhibitor NT2-31 targeted to the FAK-IGF-1R site of interaction dramatically reduced growth of melanoma, pancreatic and gastric cancers and show synergy with chemotherapy [22, 50].

Our data demonstrate that FAK and VEGFR-3 proteins and their complexes are a great target because they are present in tumor and stroma and their inhibition will affect signaling in tumor and its microenvironment. These results demonstrated that targeting the scaffolding function of FAK with the small-molecule inhibitors can be effectively used to develop potential oral-based cancer therapeutics.

## MATERIALS AND METHODS

### Cell lines

MiaPaCa-2 and Panc-1 cells were purchased from American Type Culture Collection (ATCC, Rockville, MD, USA). Cells were maintained in RPMI-1640 with 10% fetal bovine serum. All cell lines were incubated at 37°C in 5% CO_2_.

### Antibodies and reagents

VEGFR-3 and p-VEGFR-3 rabbit polyclonal antibody from Cell Aplications, Inc. and Santa Cruz Biotechnology, Inc. (Santa Cruz, CA, USA). Cell Signaling Technology (Danvers, MA, USA): Pro-caspase-8, Erk 1/2, p-Erk, Akt, p-Akt, PARP. FAK mouse monoclonal antibody (clone 4.47), Paxillin, phospho-tyrosin 4G10, VEGFR-3 clone 9D9 (Millipore, Billerica, MA). Compound C4 - Chloropyramine hydrochloride, Sigma #1915. FAK kinase inhibitor PF-562271 (Santa Cruz, CA, USA).

### Assays of cell viability

Cell survival was assayed by measuring mitochondrial dehydrogenase activity with CellTiter 96® Aqueous One Solution Cell Proliferation Assay (Promega, Madison, WI, USA) according to the manufacturer's protocol.

Detection of apoptosis was performed by TUNEL assay APO-DIRECT kit (Millipore) according to the manufacturer's recommendations. Quantitative analysis of apoptosis was performed using FlowJo program (Tree Star, Ashland, OR).

### Cell Cycle Analysis

For cell-cycle analysis, pancreatic cells were grown to 70% confluency in 100-mm plates and then serum starved for 48 hours to allow for synchronization. After 48 hours, medium was aspirated and fresh medium with C4 or vehicle was added for 24 hours. Treated medium was then collected, monolayers were washed with cold PBS, cells were trypsinized, and cell pellets were collected. Cell pellets were washed twice with PBS, fixed in cold methanol, and rewashed with PBS to remove methanol. After resuspension in 300–500 μL PBS, cells were digested with 20 μg/ml RNase and cellular DNA was stained with propidium iodide (50 μg/ml) by 3-hour incubation at room temperature in the dark. The DNA content of labeled cells was acquired using FACSCaliber cytometry (BD Biosciences, San Jose, USA) and FlowJo software (Tree Star Inc., Ashland, OR).

### Western Blot Analysis

Appropriately treated or non-treated cells were allowed to grow until they are 80-85% confluent or until treatment was completed. Cells were twice washed with ice-cold phosphate-buffered saline (PBS), then incubated on ice for 30 minutes with 1% NP-40 lysis buffer, with inhibitors as previously published [39].

### Animal models

In accordance with the RPCI IACUC approved protocol, 10^6^ cells (100 μl) were subcutaneously injected into the right flank of the 6-week old SCID mice, 5-8 in each group. Treatment with compound C4 was started the next day, after cells injection or when tumor reached approximately 100 mm^3^. Tumor size was measured twice weekly and volume was calculated using the formula length X width^2^ X 0.5. Animals were sacrificed after 21 days of treatment or when tumor size reached protocol end point. Tumor was excised, measured and preserved for protein and RNA preparation and cytochemistry.

#### Surgical orthotopic implantation

Mice were anesthetized and the abdomen was cleaned with isopropyl alcohol, and a left upper transverse incision was made to and including the peritoneum. The pancreas was exposed, and 1 × 10^6^ cells MiaPaCa-2 or cells derived from patient's tumor 11424, previously characterized in Dr. Repasky lab, were suspended in 50 μL of PBS: Matrigel were slowly injected into the body of the pancreas. Mice were allocated to 1 of 4 groups (n = 6 mice per group). The pancreas was returned, and the abdomen was closed with 5-0 Vicryl (Ethicon, Somerville, NJ). The mice were observed for 6-10 weeks and Necropsy was performed. Animals were housed in facilities approved by the American Association for Accreditation of Laboratory Animal Care and in accordance with current regulations and standards of the U.S. Department of Agriculture.

### Magnetic resonance imaging

MRI studies were conducted using a 4.7-T/33-cm horizontal bore magnet (GE NMR Instruments, Fremont, CA) incorporating AVANCE digital electronics (Bruker Medical Inc., Billerica, MA) generating maximum field strength of 950 mT/m and a custom-designed 35-mm RF transmit-receive coil. Induction of anesthesia before imaging and maintenance of anesthesia during imaging was achieved by inhalation of isoflurane (~2–3% in oxygen). Anesthetized animals were placed on an acrylic sled equipped with respiratory and temperature sensors and positioned within the magnet. An air heater system was used to maintain animal temperature in conjunction with the sensors embedded within the sled, which provided continuous feedback during imaging. Preliminary scout images were acquired on the sagittal and axial planes to assist in slice prescription for subsequent scans. T2-weighted images were acquired on the coronal plane with the following parameters: TE/TR = 41/2,500 ms, matrix size 256 × 192, 1 mm thick slices, FOV 3.2 × 3.2 cm, NEX = 4. Image processing and analysis were carried out using commercially available software (AnalyzePC; AnalyzeDirect).). A primary tumor volume (mm3) was calculated from manually traced regions-of-interest (ROI) on multislice T2-weighted images.

### Immunohistochemistry and scoring

All staining procedures were done as previously described [50]. For detection, we used Vectastain Elite ABC kit (Vector Laboratories). Diaminobenzidine (DAB) was used as the chromogen, and the slides were counterstained with hematoxylin. A negative and positive control was included in each staining. IHC-stained tissue slides were scanned in an Aperio ScanScope CS, viewed using ImageScope software, and quantified using Aperio Image Analysis algorithms (Aperio Technologies, Inc., Vista, CA). A full time research pathologist (W.B.) analyzed tissue section for Ki67 and CD31 expression. To obtain relative staining levels, five fields/samples containing a minimum of 100 cells each were analyzed for each stain. Staining analysis for Ki67 expression was done using nuclear algorithm (V9) that measured intensity (0, none; 1, weak; 2, moderate; 3, strong) and percentage of positive cells (0–100). Staining analysis for CD31 expression was done using the microvessel algorithms (v9) which reported scores for vessel density, mean for vessel lumen, mean for vessel size, average vessel thickness. Both algorithms were modified to and parameters were adjusted to enhance the performance and accuracy of the analysis. The results of the analysis were exported as CSV file format.

### Statistical analysis

For all experiments comparison between groups were made using a two-tailed two-sample Student's t test. Differences for which P value was less than 0.05 were considered statistically significant.

## Supplemental Figures


