# Supplementary material for: The FAK scaffold inhibitor C4 disrupts FAK-VEGFR-3 signaling and inhibits pancreatic cancer growth
Source: Oncotarget. 2013 Sep 30;4(10):1632–46. doi: 10.18632/oncotarget.1365 (PMC3858551; doi:10.18632/oncotarget.1365)
Supplement: Supplementary file 1 [file oncotarget-04-1632-s001.doc]

**SUPPLEMENTARY FIGURES**

**Figure S1. Specificity of FAK inhibitor C4.** The specificity of binding C4 to FAT domain of FAK was confirmed in a bio-layer interferometry binding experiment with the use of Forte-Bio Octet Red platform. We compared binding of the FAT domain of FAK with compounds C4, one of the derivatives of C4, without biological activity C4-1 and structurally related antihistamine diphenhydramine. Only C4 was binding to FAT domain with moderate affinity, K_d_=5.7 x 10^-7^ M

**Figure S2. Kinase assay with inhibitor C4.**

Invitrogen's SelectScreen® Kinase Profiling Service: Single Point analysis was performed at

100µM ATP and 1 µM C4.
